# Mortality and Years of Life Lost Due to Brain and Other Central Nervous System Cancer in Wuhan, China, from 2010 to 2019

**DOI:** 10.3390/ijerph20043544

**Published:** 2023-02-17

**Authors:** Jiahao Chen, Yan Liu, Haoyu Wen, Yaqiong Yan, Niannian Yang, Yan Guo, Juan Dai, Chuanhua Yu

**Affiliations:** 1 Department of Epidemiology and Biostatistics, School of Public Health, Wuhan University, Wuhan 430071, China; 2 Wuhan Center for Disease Control & Prevention, Wuhan 430022, China

**Keywords:** brain and other central nervous system cancer, disease burden

## Abstract

Background: Brain cancer is one of the worst types of cancer worldwide. Understanding the epidemiology of CNS cancer is critical for properly allocating healthcare resources. Methods: We collected data on CNS cancer deaths in Wuhan, China, during 2010–2019. We constructed the cause-eliminated life tables to calculate life expectancy (LE), mortality, and years of life lost (YLLs) by age and sex. The BAPC model was used to forecast the future trends of age-standardized mortality rate (ASMR). Decomposition analysis was adopted to explore the contribution of population growth, population aging, and age-specific mortality to the change in total CNS cancer deaths. Results: In 2019, the ASMR of CNS cancer was 3.75, and the ASYR was 135.70 in Wuhan, China. ASMR was expected to decrease to 3.43 in 2024. The age distribution of deaths due to CNS cancer was concentrated in the middle-aged and older population, with a peak in the 65–69 age group. Caidian, Jianghan, and Qingshan had the greatest ASMRs in 2019 in Wuhan, with ASMRs of 6.32, 4.78, and 4.75, respectively. Population aging is critical to the change in total CNS cancer deaths. Conclusion: We analyzed the current status, temporal trends, and gender and age distributions of the burden of CNS cancer in Wuhan, during 2010–2019, providing a valuable reference for better lessening the CNS cancer burden.

## 1. Introduction

Brain and other central nervous system cancer (abbreviated as CNS cancer in this article) is a group of rare and heterogeneous tumors, with over 90% of the damage happening in the brain and the remainder occurring in the meninges, spinal cord, and cranial nerves [1]. According to the 2019 Global Burden of Disease (GBD) study [2], the number of CNS cancer patients worldwide is estimated at around 1 million, with a rate of 13.77 (1/100,000), accounting for 1% of all diseases. Although CNS cancer is relatively uncommon worldwide, it accounts for a disproportionate burden of cancer mortality due to its high fatality rate. Only one-third of people survive at least 5 years after being diagnosed [3]. Moreover, the effective treatment of CNS cancer requires highly specialized and multimodal medical care, exacerbating the discrepancies in clinical outcomes between countries at different levels of development [4,5].

As the largest developing country in the world, China has undergone extensive progress in economic growth and health promotion during the past decades. The cancer spectrum has significantly shifted as a result of the lifestyle, age, and living situation changes in the Chinese population [6,7]. In 2019, China had the highest number of CNS cancer incident cases worldwide, and the number of CNS cancer deaths in China was also among the top 10 [8]. The World Health Organization (WHO) reported around 80 thousand newly diagnosed CNS cancer cases and 65 thousand deaths in China in 2020, posing a significant healthcare burden in the country [9].

Wuhan is the capital of Hubei Province, located in central China (113°41′ E–115°05′ E, 29°50′ N–31°22′ N). Wuhan has 13 counties/districts, a total size of 85,691,500 square kilometers, and a population of 13,648,900 (84.31% urban and 7.24% rural). Thoroughly assessing the CNS cancer burden in Wuhan will assist in better comprehending the gap between present circumstances and expectations and provide valuable real-world data for updating the status of the CNS cancer epidemic. In this study, we collected CNS cancer death data from the Disease Surveillance Points System (DSPs) approved by the Wuhan Center for Disease Control and Prevention. Based on these quality-controlled and low under-reporting rate data, we examined the present status, temporal trends, and gender and age distributions of the burden of CNS cancer in Wuhan [10]. This research is by far the most comprehensive and accurate assessment of the CNS cancer disease burden in Wuhan. This study’s mortality, years of life lost (YLLs), life expectancy (LE), and cause-eliminated life expectancy (CELE) metrics are critical in supporting future research.

## 2. Methods

### 2.1. Study Area and Data Sources

For this study, data on CNS cancer mortality were gathered from the Disease Surveillance Points System (DSPs), a city-wide surveillance system that covers almost 100% of Wuhan’s population. All-cause deaths occurring in the area under the jurisdiction of Wuhan are registered and reported through this surveillance system by the local health service institutions and level 2 or above hospitals. The Population Division of the Wuhan Public Security Bureau supplied population data. According to the International Classification of Diseases (ICD) tenth revision, CNS cancer coded as C70.0-C72.9 (C70, malignant neoplasm of meninges; C71, malignant neoplasm of brain; C72, malignant neoplasm of spinal cord, cranial nerves, and other areas of the CNS). Supplementary Table S1 shows the types of CNS cancer and their percentages between 2010 and 2019 in Wuhan.

### 2.2. Study Metrics

Mortality, life expectancy (LE), and cause-eliminated life expectancy (CELE) were calculated using the abbreviated life table and cause-eliminated life table [11]. Cause-eliminated life expectancy highlights the severity of a certain cause of death’s influence on population health [12]. Years of life lost (YLLs) is the number of years death happened earlier than expected. It is regarded as a precise indication of disease burden and has been broadly applied worldwide to identify and prioritize causes of premature mortality [13,14]. We calculate YLLs by multiplying the deaths at each age group by their standard life expectancy. The standard life expectancy at birth is 87.89 years for both sexes, based on the lowest observed mortality rates for each 5-year age group in populations greater than 5 million [15]. The core equation can be written as follows:

$$YLLs=\overset{\infty }{\underset{a=0}{\sum }}N\times L$$

where N is the number of death (gender-age specified), L is the standard life expectancy (age specified), and a is the age group, starting with 0–1 years, then 1–5 years, 5–10 years, and so on.

### 2.3. Decomposition of Changes in Deaths

We decomposed the change in total death tolls by year in Wuhan from 2010 to 2019 into three components: change owing to overall population growth, change due to population aging, and change in age-specific mortality. The net change in these three components equals the total death toll. The decomposition analysis method was developed in demographic research by Das Gupta [16] and has been widely used by many disease burden researchers [17,18,19]. In brief, change due to population growth was calculated by the product of population growth and mortality rate in 2010, i.e., it hypothesizes that the mortality was the same in 2019 as in 2010; thus, the estimated deaths were exclusively generated from population growth. The change due to population aging and change in age-specific and cause-specific mortality were calculated based on the equation as follows:

$$diff=\overset{\infty }{\underset{a=0}{\sum }}\left(C^{t_{2}}-C^{t_{1}}\right)\left(\frac{M^{t_{2}}-M^{t_{1}}}{2}\right)+\overset{\infty }{\underset{a=0}{\sum }}\left(M^{t_{2}}-M^{t_{1}}\right)\left(\frac{C^{t_{2}}-C^{t_{1}}}{2}\right)$$

where diff is the difference between the crude mortality in 2010 and 2019; C is age-specific population (population structure); M is age-specific mortality; $t_{1}$ and $t_{2}$ is the different period of our population (2010 and 2019 in this case); and a is the age group, starting with 0–1 years, then 1–5 years, 5–10 years, and so on. The first term on the right side of the equation is the change due to population aging, and the second term is the change in age-specific mortality. 

### 2.4. BAPC Model

A Bayesian age-period-cohort (BAPC) model was applied to predict the mortality and YLLs rate of brain and CNS cancer to 2024. This model has been described previously. In brief, the BAPC model applies a second-order random walk (RW2) to smooth out age, period, and cohort effects for a priori mortality rate and uses this to predict posterior mortality rate. To solve the convergence problem associated with the Monte Carlo sampling method employed in the traditional Bayesian methodology, the BAPC model uses the integrated nested Laplace approximation (INLA) to estimate the marginal posterior distribution, resulting in more accurate findings. BAPC and INLA packages in R software implemented the analyses and data visualization.

### 2.5. Statistical Analysis

Age-standardized rates were calculated using the age standard from the Sixth National Population Census. The age-standardized mortality rate (ASMR) calculation formula is as follows:

$$ASMRs=\frac{\sum^{\;}\begin{array}{c} Age\;composition\;of\;standard\;group\;population\\ \times Age\;specific\;mortality \end{array}}{Age\;composition\;of\;standard\;population}$$

The age-standardized rate of YLLs (ASYR) calculation stays the same as ASMR. All rates are given in terms of 100,000 people or person-years. Furthermore, the estimated annual percentage change (EAPC) was calculated to characterize the significance of the trends from 2010 to 2019: a generalized linear model, lnR = α + βT + ϵ, was fitted, where R is the count or rate and T is the calendar year. EAPC was calculated as 100 (exp[β]—1), with a 95% confidence interval (CI). R software (version 3.3.3) was used to perform all data analysis.

## 3. Results

### 3.1. Temporal Trends of CNS Cancer Burden from 2010 to 2019 and the Forecast for the Next Five Years

**Figure 1:**
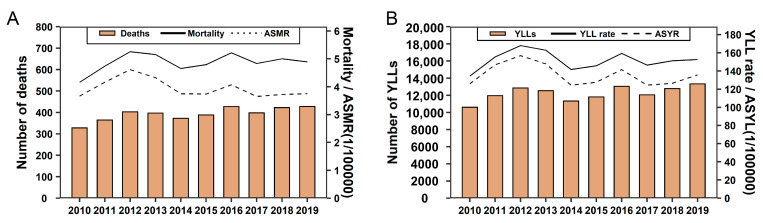
Temporal trends of CNS cancer burden from 2010 to 2019. (**A**) Temporal trends of deaths, mortality, and ASMR from 2010 to 2019. (**B**) Temporal trends of YLLs, YLL rate, and ASYR from 2010 to 2019.

Figure 1 shows the temporal trends of deaths and YLLs from 2010 to 2019 in Wuhan, alongside their age-standardized rates. In 2019, the number of CNS cancer deaths was 428, with a rate of 4.89 and ASMR of 3.75 (Figure 1A); CNS cancer caused 13331 YLLs, with a rate of 152.60 and ASYR of 135.70 (Figure 1B). Between 2010 and 2019, temporal trends of CNS cancer burden show large fluctuations over the years. Although the number of CNS cancer deaths increased by 30.4%, the mortality and ASMR did not present any significant increasing or decreasing trends, with EAPC of 0.91 (−0.79 to 2.64) and −1.17 (−3.10 to 0.80), respectively. The YLLs barely showed the same trends. The EAPC of YLLs rate and ASYR were 0.14 (−1.65 to 1.96) and −0.98 (−3.09 to 1.18), respectively.

**Figure 2:**
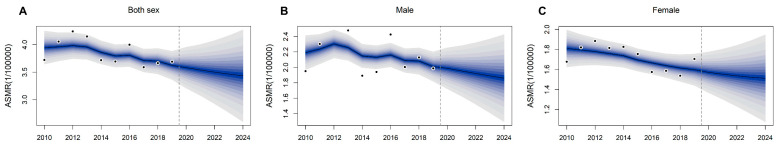
BAPC prediction of ASMR from 2010 to 2024 by gender. (**A**) the prediction result for both sex. (**B**) the prediction result for male. (**C**) the prediction result for female.

The ASMR of CNS cancer for the next five years was predicted by the BAPC model, as shown in Figure 2. For both sex (Figure 2A) and female (Figure 2C), all measured ASMR values lie in the confidence interval, which proved the effective fitting of the prediction model. The ASMR was expected to decrease to 3.43 in 2024 for both sex and 1.51 for females. 

As is shown in Supplementary Table S2, in 2019, the LE of the Wuhan population was 81.42 years (78.94 years for males and 84.08 years for females). The CELE of the Wuhan population was 81.52 years (79.04 years for males and 84.18 years for females). The LE lost due to CNS cancer was 0.10 (0.10 years for males and 0.11 years for females). The LE lost due to CNS cancer presents a stable trend between 2010 and 2019, at an average of 0.10 years, with no difference found between males and females.

### 3.2. Age and Gender Distribution of CNS Cancer Burden

**Figure 3:**
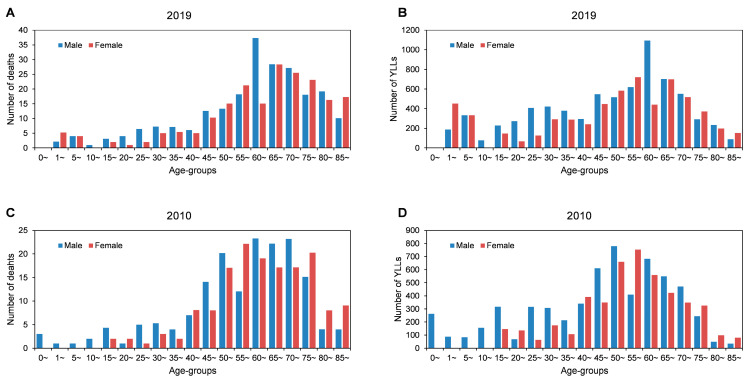
Deaths and YLLs related to CNS cancer by age and sex in 2010 and 2019. (**A**) Number of deaths in 2019. (**B**) Number of YLLs in 2019. (**C**) Number of deaths in 2010. (**D**) Number of YLLs in 2010.

Deaths and YLLs related to CNS cancer by age and sex in 2010 and 2019 are shown in Figure 3. The age distribution of deaths due to CNS cancer was concentrated in the middle-aged and elderly population, with a peak in the 65–69 age group. The median age of CNS cancer death was 64.91 years for males and 63.20 years for females. The discrepancy in the age distribution of death and YLLs between males and females was most pronounced in the 60–65-year age group. Generally, the number of deaths and YLLs was consistently higher in males than in females.

### 3.3. Spatial Distribution of CNS Cancer Burden

**Figure 4:**
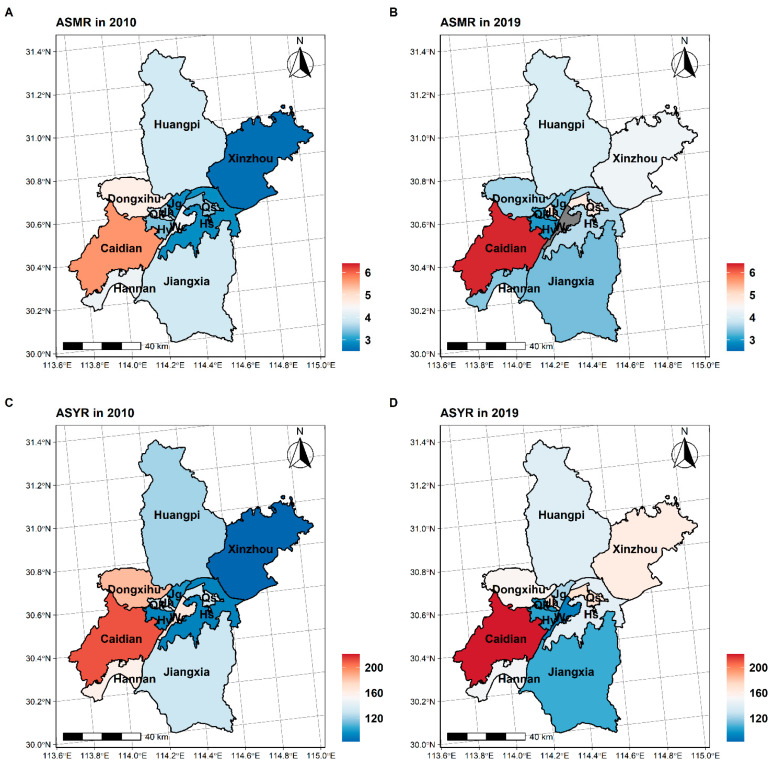
Spatial distribution of CNS cancer burden in Wuhan between 2010 and 2019. (**A**) The distribution of ASMR in 2010. (**B**) The distribution of ASMR in 2019. (**C**) The distribution of ASYR in 2010. (**D**) The distribution of ASYR in 2019.

Figure 4 shows the spatial distribution of ASMR and ASYR in Wuhan between 2010 and 2019. Both ASMR and ASYR varied considerably across different districts in Wuhan. Caidian, Jianghan, and Qingshan had the greatest ASMRs in 2019, with ASMRs of 6.32, 4.78, and 4.75, respectively. The top three districts with the highest ASYRs were the same as above, with ASYRs of 220.27, 169.51, and 168.13, respectively. From 2010 to 2019, Xinzhou has increased the most in both ASMR and ASYR, for an increasing average percentage of 81.0%. Wuchang has decreased the most in both ASMR and ASYR, with an average decreasing percentage of 45.7%.

### 3.4. Decomposition Analysis of Change in Mortality from 2010 to 2019

**Figure 5:**
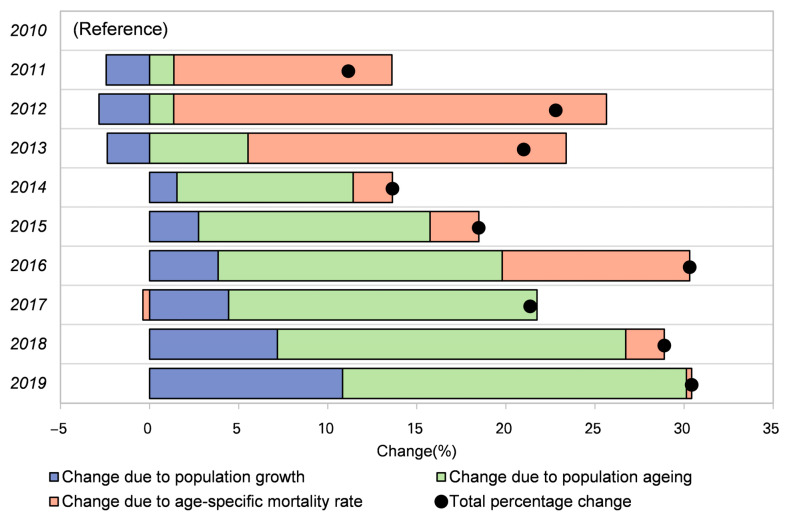
Decomposition analysis of change in mortality from 2010 to 2019.

Figure 5 shows the drivers of total percentage changes in mortality, including population growth, aging, and changes due to age-specific mortality rate, which varied substantially by year in Wuhan from 2010 to 2019. Total percentage changes in mortality increased from 11.16% in 2010 to 30.43% in 2019, with large fluctuations over the years. Of note, the change due to population aging shows a steady upward trend. In 2010, the change due to population aging only consisted of 1.36% in total percentage change. In 2019, this percentage increased to 19.3%, indicating a critically important role of population aging in CNS cancer burden.

## 4. Discussion

We comprehensively analyzed the current status, temporal trends, and gender and age distributions of the burden of CNS cancer in Wuhan from 2010 to 2019. A BAPC model was adopted to predict the ASMR and ASYR change in the next five years. Moreover, death decomposition analysis was conducted to analyze the drivers of change in CNS cancer deaths by years in Wuhan. Generally, our study provides an updated overview of the CNS cancer epidemic.

In 2019, there were 428 deaths due to CNS cancer in Wuhan, with an ASMR of 3.75. CNS cancer caused 13,331 YLLs, with an ASYR of 135.70. Temporal trends of both metrics have fluctuated over the years, showing a non-significant upward or downward trend. However, the prediction model posts positive downward trends of the ASMR in the next five years, which will be 3.43 in 2024. According to GBD 2019, the global mortality and YLLs rates caused by CNS cancer were 3.18 and 110.25, significantly lower than our results. Another research scoped at the national level indicated that in 2019, there were approximately 63527 deaths in China, with an ASMR of 3.5 (2.62–4.21), which decreased by 9.6% from 1990 to 2019 [20]. In recent years, studies on the local burden of CNS cancer in other areas of China have been scarce. A similar study based on a cancer registration system conducted in Wuwei, a northwestern city in China, from 2003 to 2012 reported a CNS cancer mortality rate of 2.64 [21]. Another study conducted in Hebei Province reported a CNS cancer mortality rate of 2.65 [22]. Both of these rates were lower than our findings, partly due to improvements in CNS cancer registries and advances in diagnostic technology and partly due to the impact of socioeconomic position (SEP) on CNS cancer risk. Evidence from several studies [23,24] suggests that higher SEP is associated with an increased risk of adult CNS cancer compared to individuals with lower SEP. The CBTRUS statistical report [25] showed an average annual age-adjusted mortality rate of 4.43 (4.40–4.46) in the US during 2014–2018, significantly higher than our findings, reinforcing the above point.

Our research found substantial gender variations in the prevalence of CNS cancer in Wuhan. Both ASR of mortality and YLL rates were higher in males than females. Some previous studies have confirmed the prolonged survival of females with CNS cancer than males [26]. Males have been found to be more susceptible to astrocytomas, notably primary glioblastoma (GBM), the most aggressive form of astrocytoma [27], although meningioma (the most common non-malignant tumor) was found to be more common in females [26]. For age distribution, our study indicated that CNS cancer deaths mainly concentrated in the middle-aged and elderly, with some in the younger age group. Childhood CNS cancers were the most prevalent pediatric solid tumors, accounting for the most mortality and morbidity in children [28,29]. On the other hand, CNS cancer, like most other neoplasms, had the highest mortality rate among the older population [30,31]. The decomposition analysis of total death tolls by region broken down into changes due to population growth, population aging, and changes in age-specific and cause-specific mortality also highlights the critical role of aging in changing CNS cancer deaths. According to the Seventh National Census data, in 1990, China’s elderly population (≥65 years old) accounted for 5.57%. By 2010, the elderly population had increased to 8.87%, and in 2020, the elderly population accounted for 13.50%. China is accelerating into an aging society [32]. The growing problem of an aging population may further increase the burden of CNS cancer in the future. 

There were significant differences in the distribution of the burden of CNS cancer among jurisdictions under Wuhan. The remote areas in the east and west had higher ASR of mortality and ASR of YLL than the central regions, especially Caidian and Xinzhou. However, even in the central city with better economic development and more hospitals, Hannan and Qiaokou districts still have a high ASR of mortality and YLL rates. It suggests that regional variability in the burden of CNS cancer may not be directly related to socioeconomic development, consistent with some previous studies [33]. These findings suggested that factors other than sociodemographic development may account for the marked regional differences in the disease burden of CNS cancer. 

According to the CBTRUS report [25], the most common of all malignant CNS tumors was glioblastoma (48.6%) with the highest incidence rate of (3.23), followed by malignant glioma, NOS (not otherwise specified) (0.51), diffuse astrocytoma (0.45), and lymphoma (0.43). As shown in Supplementary Table S1 and Figure S1, CNS cancers were divided into three major parts according to their anatomical locations. From 2010 to 2019, malignant neoplasm of brain accounted for the majority of CNS cancer deaths (88.93%), followed by malignant neoplasm of meninges (7.30%), and malignant neoplasm of spinal cord, cranial nerves, and other parts of the central nervous system (3.77%). The identified malignant tumor sites were mainly concentrated in the cerebral meninges, cerebrum, and spinal cord. In addition, malignant neoplasm of the meninges is the only CNS cancer that causes more deaths in females than in males.

The strength of our study is that we have systematically quantified the disease burden of CNS cancer in Wuhan for the first time, and the output metrics are the basis for future studies. However, some limitations could not be ignored, the first being that we did not calculate disability-adjusted life years (DALYs), a commonly used indicator for assessing disease burden [34]. Although DALYs for CNS cancer was mainly driven by YLLs rather than YLDs [15], it still needs to be considered in future studies to calculate the DALYs for a more comprehensive assessment. Secondly, we aggregated all CNS cancer into a single group. Given the considerable heterogeneity in outcomes between low-grade and high-grade brain tumors, such as gliomas and meningioma [35], the analyses of brain tumors as a single group should be seen as the first step until more detailed analyses can be performed. Thirdly, surgical or adjuvant treatments would influence the outcome and overall survival of CNS cancer patients. In the future, quantitative analysis of these variables will help to improve the results further. Finally, our data were collected until December 2019, and the mortality data collection for Wuhan City in 2020 and beyond may be underinclusive or biased due to the impact of COVID-19, which should be considered in future research.

A major global health concern associated with CNS cancer is the need for highly specialized medical and surgical treatment for diagnosis and long-term management. There is no simple, population-wide screening tool that can be utilized for early, uniform diagnosis, and CNS cancer symptoms are usually non-specific and progress to life-threatening circumstances before definite radiological diagnosis. In certain ways, paying close attention to the changes in the CNS cancer burden in a specific region and conducting periodic and targeted resource allocation may be an effective way to tackle the CNS cancer pandemic.

## 5. Conclusions

Our study comprehensively analyzed the current status, temporal trends, and gender and age distributions of the burden of CNS cancer in Wuhan during 2010–2019. Providing important information about CNS cancer burden, these findings can be used by policymakers and other stakeholders to improve prevention and control of CNS cancer burden. 

## Data Availability

The datasets used and/or analyzed during the current study are available from the corresponding author upon reasonable request.

## Supplementary Materials

The following supporting information can be downloaded at: https://www.mdpi.com/article/10.3390/ijerph20043544/s1. Table S1. Total counts of deaths stratified according to ICD-10 codes of CNS cancer from 2010 to 2019 by gender. Figure S1. Age and sex distribution of the three main (anatomically located) CNS cancer. Table S2. Life expectancy, cause eliminated Life expectancy, and life expectancy lost due to CNS cancer in Wuhan, 2010–2019.
