# Supplementary material for: Mortality and Years of Life Lost Due to Brain and Other Central Nervous System Cancer in Wuhan, China, from 2010 to 2019
Source: Int J Environ Res Public Health. 2023 Feb 17;20(4):3544. doi: 10.3390/ijerph20043544 (PMC9968237; doi:10.3390/ijerph20043544)
Supplement: Supplementary file 1 [file ijerph-20-03544-s001.zip › ijerph-2058267-supplementary.pdf]

International Journal of
Environmental Research and Public Health

MDPI

Supplementary materials

Table S1. Total counts of deaths stratified according to ICD-10 codes of CNS cancer from 2010 to 2019 by gender.

ICD code	Anatomical position	Both sex	Percentage	male	Percentage	female	Percentage
Malignant neoplasm of meninges		213	7.30%	95	6.03%	118	8.79%
C70.0	Cerebral meninges	190	6.51%	84	88.42%	106	89.83%
C70.1	Spinal meninges	16	0.55%	10	10.53%	6	5.08%
C70.9	Meninges, unspecified	7	0.24%	1	1.05%	6	5.08%
Malignant neoplasm of brain		2596	88.93%	1412	89.59%	1184	88.16%
C71.0	Cerebrum	545	18.67%	307	21.74%	328	27.70%
C71.1	Frontal lobe	47	1.61%	29	2.05%	18	1.52%
C71.2	Temporal lobe	111	3.80%	66	4.67%	45	3.80%
C71.3	Parietal lobe	27	0.92%	16	1.13%	11	0.93%
C71.4	Occipital lobe	12	0.41%	8	0.57%	4	0.34%
C71.5	Cerebral ventricle	114	3.91%	63	4.46%	51	4.31%
C71.6	Cerebellum	92	3.15%	45	3.19%	47	3.97%
C71.7	Brain stem	105	3.60%	57	4.04%	48	4.05%
C71.8	Overlapping lesion	25	0.86%	16	1.13%	9	0.76%
C71.9	Brain, unspecified	1518	52.00%	805	57.01%	713	60.22%
Malignant neoplasm of spinal cord, cranial nerves and other parts of central nervous system		110	3.77%	69	4.38%	41	3.05%
C72.0	Spinal cord	55	1.88%	35	50.72%	20	48.78%
C72.1	Cauda equina	1	0.03%	1	1.45%	0	0.00%
C72.2	factory nerve	2	0.07%	2	2.90%	0	0.00%
C72.3	Optic nerve	11	0.38%	9	13.04%	2	4.88%
C72.4	Acoustic nerve	4	0.14%	2	2.90%	2	4.88%
C72.5	Other and unspecified cranial nerves	10	0.34%	6	8.70%	4	9.76%
C72.8	Overlapping lesion	3	0.10%	3	4.35%	0	0.00%
C72.9	Central nervous system, unspecified	24	0.82%	11	15.94%	13	31.71%

Figure S1. Age and sex distribution of the three main (anatomically located) CNS cancer.

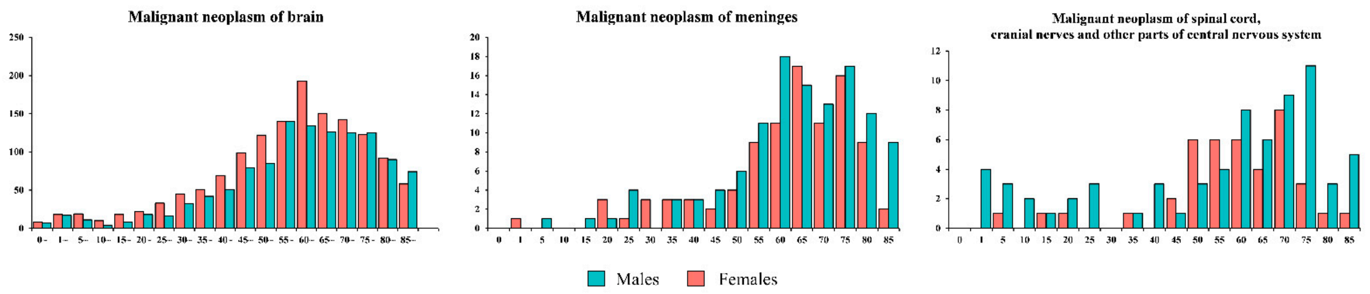

Table S2. Life expectancy, cause eliminated Life expectancy, and life expectancy lost due to CNS cancer in Wuhan, 2010-2019.

Time	Life expectancy			Cause eliminated Life expectancy			life expectancy lost due to brain tumor		
	Male	Female	All population	Male	Female	All population	Male	Female	All population
2010	77.38	82.36	79.79	77.48	82.45	79.88	0.10	0.09	0.09
2011	77.16	81.99	79.50	77.27	82.09	79.60	0.11	0.10	0.10
2012	76.34	81.09	78.64	76.46	81.19	78.74	0.11	0.09	0.10
2013	77.00	82.24	79.53	77.11	82.34	79.63	0.11	0.09	0.10
2014	78.24	83.24	80.66	78.33	83.35	80.76	0.09	0.11	0.10
2015	78.30	83.37	80.75	78.39	83.48	80.85	0.10	0.11	0.10
2016	78.43	83.67	80.95	78.55	83.77	81.06	0.12	0.09	0.11
2017	78.59	83.89	81.14	78.68	83.99	81.24	0.09	0.11	0.10
2018	78.68	83.87	81.18	78.79	83.96	81.28	0.11	0.09	0.10
2019	78.94	84.08	81.42	79.04	84.18	81.52	0.10	0.11	0.10
